# KRAS mutation as a prognostic factor in ampullary adenocarcinoma: a meta-analysis and review

**DOI:** 10.18632/oncotarget.11156

**Published:** 2016-08-09

**Authors:** Bum Jun Kim, Hyun Joo Jang, Jung Han Kim, Hyeong Su Kim, Jin Lee

**Affiliations:** ^1^ Division of Hemato-Oncology, Department of Internal Medicine, Kangnam Sacred-Heart Hospital, Hallym University Medical Center, Hallym University College of Medicine, Seoul 07441, Republic of Korea; ^2^ Division of Gastroenterology, Department of Internal Medicine, Dongtan Sacred-Heart Hospital, Hallym University Medical Center, Hallym University College of Medicine, Hwasung 18450, Republic of Korea

**Keywords:** ampullary adenocarcinoma, KRAS mutation, prognosis, meta-analysis

## Abstract

Ampullary adenocarcinoma (A-AC) is a rare malignancy arising from the ampulla of Vater. KRAS mutation is detected in 30–40% of patients with A-AC, but its clinical implication and prognostic value are not well described. We conducted this meta-analysis to investigate the association between KRAS mutation and prognosis in patients with A-AC. We searched Pubmed, MEDLINE, EMBASE, and the Cochrane Library databases for articles including following terms in their titles, abstracts, or keywords: ‘ampullary or periampullary or ampulla of vater’, ‘cancer or carcinoma’, and ‘KRAS’. There were five studies with survival data of patients. A total of 388 patients with A-AC from the 5 studies were included in the overall survival (OS) analysis, and 169 patients from 2 studies were eligible for the relapse-free-survival (RFS) analysis. Out of 388 patients, 175 (45%) had KRAS mutation. There was no association between KRAS mutation and OS (HR = 1.06, 95% CI: 0.87–1.29, *P* = 0.58). However, there was a significant correlation between KRAS mutation and worse RFS (HR = 2.74, 95% CI: 1.52–4.92, *P* = 0.0008). In conclusion, this meta-analysis indicates that KRAS mutation is associated with poor RFS, but not with OS in patients with A-AC.

## INTRODUCTION

Ampullary adenocarcinoma (A-AC) is a rare malignancy arising from the ampulla of Vater and accounts for less than 1% of all gastrointestinal cancers [1]. The ampulla of Vater is a complex region with distinct anatomic structures which include the common bile duct, pancreatic duct, and the duodenum [2]. Due to this complex coalescence of distinct structures, this small area gives rise to a heterogenous group of tumors with different prognosis [3–5].

The histological differentiation [6–8], lymph node involvement [9, 10], and vascular invasion [11] have been considered as important prognostic factors for A-AC. However, these parameters were unable to predict the prognosis of A-AC. In addition, the role of molecular and genomic profiles of A-AC as a prognostic factor has not been well investigated.

KRAS gene is included in the mammalian Ras gene family and plays a key role in Ras/mitogen-activated protein kinase signaling [12]. Somatic mutations in KRAS gene act as an early event in the carcinogenesis of human cancers. KRAS mutation is detected at high rate in lung cancer, colorectal cancer (CRC), and pancreatic cancer [13–15]. The presence of mutant KRAS in pancreatic cancer correlated with poor prognosis [16]. KRAS mutation was also associated with a lack of response to EGFR inhibitor in CRC [17]. Mutations in KRAS gene are also found in 30–40% of patients with A-AC [18–20], but their prognostic value has not been revealed.

We performed this meta-analysis of previous studies to investigate the prognostic value of KRAS mutation in patients with A-AC.

## RESULTS

### Results of search and eligible studies

**Figure 1:**
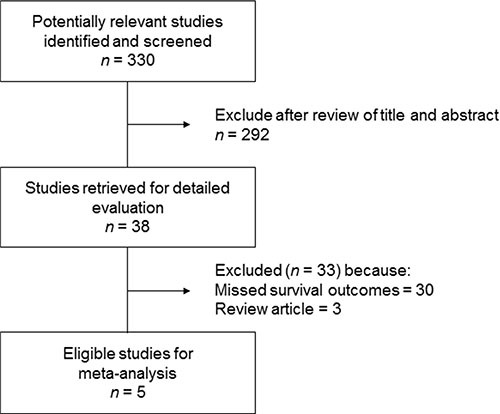
Flow diagram of search process

Figure 1 is the flowchart of studies accessed through the review process. The search process yielded 330 studies of which 38 potentially relevant studies were retrieved and assessed in detail. After excluding 33 studies (30 studies missed time-dependent survival data and 3 studies were review article), the remaining 5 studies [20–24] fulfilled our eligibility criteria and were included in the meta-analysis. A total of 388 patients were collected from the five studies (Table 1).

**Table 1:** Summary of the five studies included in the current meta-analysis

Study (ref. no.)	KRAS status	*n* (%)	RFS (months)	HR for RFS (95% CI)	Median OS (months)	HR for OS (95% CI)
Howe et al. [20]	mutant	34 (37%)	NA	NA	69.7	0.943 (0.68-1.30) *P* = 0.721
	wild	58 (63%)	NA		47.6	
Schultz et al. [21]	mutant	72 (67%)	25.0 (Median)	2.45 (1.19–5.06) *P* = 0.015	22.3	1.93 (1.12–3.31) *P* = 0.018
	wild	35 (33%)	Not reached		44.7	
Valsangkar et al. [22]	mutant	25 (33%)	NA	NA	NA	1.103 (0.76–1.61) *P* = 0.6087
	wild	50 (67%)	NA		NA	
Mikhitarian et al. [23]	mutant	25 (48%)	NA	NA	45	0.918 (0.65–1.30) *P* = 0.631
	wild	27 (52%)	NA		44	
Kwon et al. [24]	mutant	19 (31%)	29 (Mean)	3.384 (1.25–9.20) *P* = 0.017	NA	1.060 (0.51-2.22) *P* = 0.877
	wild	43 (69%)	98 (Mean)		NA	

HR, hazard ratio; RFS, relapse-free-survival; OS, overall survival; NA; not available.

### KRAS mutation

The incidence of KRAS mutation was various from 30% to 67% among the five studies. Out of 388 patients, 175 (45%) had KRAS mutation. Of 175 patients with mutant KRAS, 134 (76.5%) had mutation at codon 12 and the most common mutation types were G12D and G12V.

### KRAS mutation and overall survival

**Figure 2:**
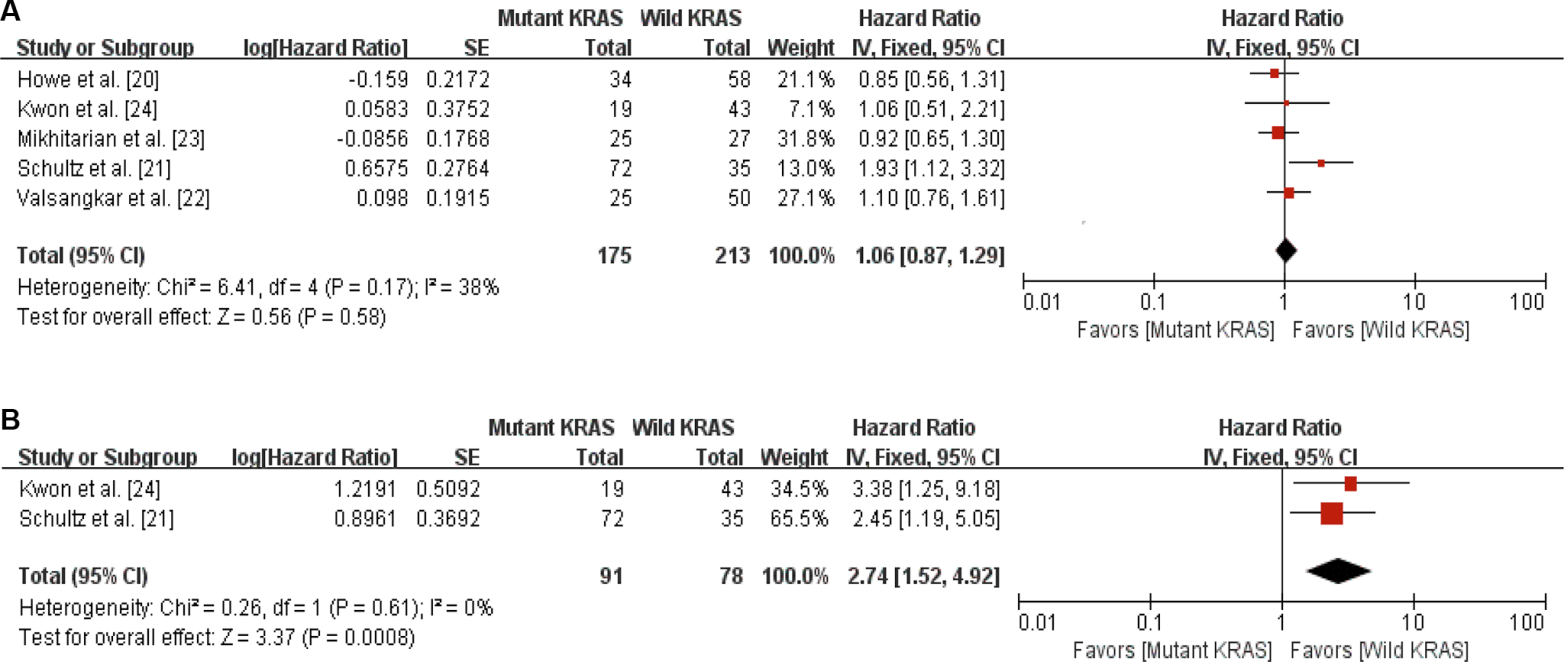
Forest plots for the relation between KRAS mutation and survival outcomes (**A**) The relation between KRAS mutation and overall survival. (**B**) The relation between KRAS mutations and relapse-free survival.

We pooled the survival data from the 5 studies to evaluate the association of KRAS mutation and overall survival (OS) in patients with A-AC. Two of 5 studies [21, 24] presented hazard ratio (HR) and 95% confidence interval (CI) in the article. In the remaining 3 studies [20, 22, 23], we calculated HR and 95% CI from the available data. As shown in Figure 2A, there was no association between KRAS mutation and OS (HR = 1.06, 95% CI: 0.87–1.29, *P* = 0.58). There was no statistical heterogeneity among the studies.

### KRAS mutation and relapse-free-survival

Of the 5 studies, only 2 studies [21, 24] reported relapse-free survival (RFS). A total of 169 patients from the 2 studies were included in the RFS analysis. As shown in Figure 2B, there was a significant association between KRAS mutation and worse RFS in patients with A-AC (HR = 2.74, 95% CI: 1.52–4.92, *P* = 0.0008).

## DISCUSSION

Because A-ACs are originated from three distinct epithelium (duodenal, biliary, pancreatic) of the ampulla of Vater, it is unclear whether A-ACs have homogenous or heterogenous prognosis. To set up the effective treatment strategy in this type of cancer, it is important to classify the subgroup according to the prognosis. Because of its rarity, however, prognostic factors are not well established.

A number of studies have attempted to classify the disease by identifying prognostic factors. The first approach was to divide the patients as either intestinal-type or pancreatobiliary-type by histology. Several studies found that pancreatobiliary-type showed worse outcome [6, 25, 26]. The second approach was to investigate markers to identify distinct prognostic subgroups of A-ACs. Several markers including intestinal-specific markers, cytokeratin, or microsatellite instability have been evaluated, but the results were limited by small sample size [5, 6, 8, 27–30]. Recently, molecular analysis seems to be a promising approach to identify prognostic factors. Overman et al. segregated A-ACs into two subgroups, intestinal-like subgroup and biliary-like subgroup by gene expression profile, and found that the expression of CK7+/CK20− was dominant in biliary-like subgroup and associated with poor prognosis [7]. In this study, activation of the PI3K-AKT and RAS-RAF-MAPK pathway were also increased in the poor prognostic biliary-like subgroup.

Mutations in KRAS gene are known to be detected in 30–40% of patients with A-AC [15–17]. In this meta-analysis, 175 (45%) out of 388 patients had KRAS mutation. Most KRAS mutation was located at codon 12 and the most common mutation types were G12D and G12V. The association between KRAS mutation and histological subtype was analyzed in three of the five studies and there was no difference in the incidence of KRAS mutation between intestinal-type and pancreatobiliary-type [20, 23, 24]. In one study, KRAS mutation was more frequently detected in poorly differentiated tumors than well-differentiated tumors [24].

The role of KRAS mutation as a prognostic factor is controversial in lung cancer [31–33]. However, KRAS mutation at codon 12 or 13 was associated with worse prognosis in CRC [34, 35]. In pancreatic cancer, recent meta-analysis demonstrated that KRAS mutation was a potential poor prognostic marker [16]. In patients with A-AC, however, its prognostic value has not been revealed. Of the five studies included in our meta-analysis, two evaluated the association between KRAS mutation and RFS [22, 24]. In both studies, KRAS mutation was associated with poor RFS. Our meta-analysis with 169 patients from the two studies demonstrated that there was a significant correlation between KRAS mutation and worse RFS (HR = 2.74, 95% CI: 1.52–4.92, *P* = 0.0008). In terms of OS, only one study found that KRAS mutation was associated with poor OS [21]. In another study, subgroup analysis showed that patients with KRAS-G12D mutation had poor OS, compared to patients with wild-type KRAS [22]. In our meta-analysis with 388 patients, KRAS mutation was not associated with OS (HR = 1.06, 95% CI: 0.87–1.29, *P* = 0.58). Considering that KRAS mutation related to shorter RFS, however, KRAS mutation might be a potential survival factor in patients with A-AC. Therefore, further studies incorporating detailed subgroup analysis with large population are needed to reveal the relationship between KRAS mutation and survival in patients with A-AC.

This study has several limitations. First, the small number of studies was included in this meta-analysis, with 5 studies for OS analysis and 2 studies for RFS analysis. Second, all the five studies were retrospective review and important clinical information including adjuvant treatment after surgery and palliative chemotherapy which might affect the RFS and OS were not presented. Lastly, because KRAS mutation was not classified into mutational subgroups in most studies, we could not perform subgroup analysis.

In conclusion, this meta-analysis indicates that KRAS mutation is associated with poor RFS, but not with OS in patients with A-AC. Considering that small number patients were included and anti-cancer treatments after surgery could not be analyzed in this study, however, further studies with large population are still needed to reveal the relationship between KRAS mutation and prognosis in patients with A-AC.

## MATERIALS AND METHODS

### Searching strategy

We searched Pubmed, MEDLINE, EMBASE and the Cochrane Library databases (up to May 2016) for articles that included the following medical terms in their titles, abstracts, or keyword lists: ‘ampullary or periampullary or ampulla of vater’, ‘cancer or neoplasm or carcinoma or malignancy’, ‘KRAS’ or ‘K-ras’, ‘prognosis or survival’. All eligible studies were retrieved, and their bibliographies were checked for other relevant publications. Review articles and bibliographies of other relevant studies identified were hand searched to find additional eligible studies. Additionally, we searched all abstracts and virtual meeting presentations from the American Society of Clinical Oncology (ASCO) conferences held between 2007 and 2016 and sought expert opinion to identify relevant but unpublished studies.

### Inclusion criteria

Clinical studies that met the following inclusion criteria were included in the meta-analysis: (i) all patients diagnosed of ampullary cancer were confirmed through histopathologic detection; (ii) KRAS mutational status was accessed by extracting genomic DNA; (iii) the relationship between KRAS mutation and the prognosis of patients with ampullary cancer was investigated by measuring time-dependent end-point, including RFS or OS; (iv) HR for time-dependent end-points were reported or could be calculated from the data provided.

### Data extraction

Data were carefully extracted from all eligible studies by two of the authors (BJK and JHK) independently, and discrepancies were resolved by consensus including a third author (HJJ). The following data were collected from each study: first author's name, year of publication, number of patients, mutation rates for KRAS, time-dependent endpoint including RFS and OS, and HR for time-dependent endpoint.

### Statistical analysis

The association between KRAS mutation and RFS or OS was presented as HR with 95% CI. The HR and 95% CI as relevant effect measures were estimated directly or indirectly from the given data. A fixed effect model was used to calculate the pooled HR estimate. HRs for death were combined using an inverse variance method based on a logarithmic conversion; 95% CIs were used to determine the standard error (SE) using the formula SE = 95% CI/1.96. The traditional *Q*-test and the *I*^2^ statistic were used to evaluate heterogeneity. Significant heterogeneity was considered to be present for *P* < 0.05 in the *Q* test or for I2 > 30%. The *Z*-test for overall effect and its two-sided *P*-value were also assessed. RevMan v5.2 software was used to report outcomes.
